# Evaluation of solvent effect on the extraction of phenolic compounds and antioxidant capacities from the berries: application of principal component analysis

**DOI:** 10.1186/s13065-014-0048-1

**Published:** 2014-08-22

**Authors:** Joana Schuelter Boeing, Érica Oliveira Barizão, Beatriz Costa e Silva, Paula Fernandes Montanher, Vitor de Cinque Almeida, Jesuí Vergilio Visentainer

**Affiliations:** Departament of Chemistry, State University of Maringa, Maringa, Parana 87020-900 Brazil; Institute of Chemistry, State University Paulista, Araraquara, Sao Paulo 14800-060 Brazil

**Keywords:** Solvent extraction, *Morus nigra*, *Rubus ulmifolius*, *Fragaria* x *ananassa*, Anthocyanins, Multivariate analysis

## Abstract

**Background:**

This study evaluated the effect of the solvent on the extraction of antioxidant compounds from black mulberry (*Morus nigra*), blackberry (*Rubus ulmifolius*) and strawberry (*Fragaria* x *ananassa*). Different extracts of each berry were evaluated from the determination of total phenolic content, anthocyanin content and antioxidant capacity, and data were applied to the principal component analysis (PCA) to gain an overview of the effect of the solvent in extraction method.

**Results:**

For all the berries analyzed, acetone/water (70/30, v/v) solvent mixture was more efficient solvent in the extracting of phenolic compounds, and methanol/water/acetic acid (70/29.5/0.5, v/v/v) showed the best values for anthocyanin content. Mixtures of ethanol/water (50/50, v/v), acetone water/acetic acid (70/29.5/0.5, v/v/v) and acetone/water (50/50, v/v) presented the highest antioxidant capacities for black mulberries, blackberries and strawberries, respectively.

**Conclusion:**

Antioxidants extractions are extremely affected by the solvent combination used. In addition, the obtained extracts with the organic solvent-water mixtures were distinguished from the extracts obtained with pure organic solvents, through the PCA analysis.

**Electronic supplementary material:**

The online version of this article (doi:10.1186/s13065-014-0048-1) contains supplementary material, which is available to authorized users.

## Background

Berry fruits such as blackberry (*Rubus ulmifolius*), black mulberry (*Morus nigra*) and strawberry (*Fragaria* x *ananassa*) are one of the richest source of antioxidants and phytochemicals among fruits and vegetables [1–3]. They are widely recognized due to their several health-promoting properties, including the reduced risks of cancer, obesity, cardiovascular disease and other chronic diseases [4–6]. These beneficial functions have been attributed to polyphenolic compounds such as anthocyanins [3,4,7].

Extraction is the first step in analysis polyphenolic, which consists in isolation of phenolic compounds from plant materials. So, the used method in this procedure becomes essential for the accurate quantification and determination of antioxidant capacity [8]. Several extraction conditions are reported in the literature, however there is no single extraction method which may be considered standard [9,10]. The chemical nature of phenolic compounds, the extraction method employed, the sample particle size, storage time and conditions, as well as the presence of interfering substances affect the efficiency of the extraction methods [11].

Solid–liquid extraction method of phenolic compounds with different solvents from vegetable sources are the most commonly used for isolating these compounds [12,13]. Crude phenolic extracts contain complex mixtures of some classes of phenols, which are selectively soluble in the different solvents. In this sense, solvent polarity plays a key role in increasing phenol solubility [14].

Principal component analysis (PCA) is a multivariate data analysis whose main aim is to represent a large set of data through limited multivariate data, called principal components (PCs) [15]. Thus, it is possible to reduce the dimensionality of a data set while preserving the maximum information. The PCA may reveal groups of observations, trends, and outliers. Furthermore it also can uncover the relationships between observations and variables and between the variables themselves [16,17]. In turn, the PCA has been widely applied in diverse areas of investigation [18–20,15,21,22].

This work is the first attempt to identify the best solvents in the extraction of antioxidant compounds from three different berries cultivated in southern Brazil. For this, various antioxidant capacity assays were performed: FRAP, DPPH^•^ and ORAC. In addition, total phenolic content and anthocyanin content were determined. Also, principal component analysis (PCA) has been carried out to study the influence of the extraction procedure on the antioxidant compounds of the berry extracts. This research serves as a good basis for other researchers to investigate berry antioxidants in future research.

## Results and discussion

### Total phenolic content and total anthocyanin content

The total phenolic contents (TPC) and total anthocyanin contents (ACC) of the berry extracts are shown in Additional file 1. According to the results, TPC ranged from 116.47 ± 2.07 to 5744.55 ± 20.69 g GAE/ kg DW for the black mulberry, 479.28 ± 3.30 to 4280.93 ± 28.08 g GAE/ kg DW for the blackberry and 480.72 ± 5.48 to 2958.05 ± 18.67 g GAE/ kg DW for the strawberry. The ACC of berry extracts by the pH differential method ranged from 739.15 ± 0.00 to 3692.19 ± 8.51 for black mulberry, from 308.04 ± 14.00 to 754.79 ± 10.56 for blackberry and from 167.24 ± 0.82 to 349.08 ± 2.42 g CGE/ kg for strawberry.

Among the solvents used, extraction with acetone/water (70/30, v/v) showed the highest value of TPC for black mulberry and blackberry extracts. For strawberry extracts, the highest TPC were obtained for acetone/water (50/50, v/v) and (70/30, v/v) extraction solution, which no showed significant differences between them (p < 0.05). The lowest TPC for all fruits was obtained using acetone and acetone/acetic acid (99.5/0.5, v/v) mixture solution. The TPC of the black mulberry was significantly higher than that of the blackberry and strawberry.

According to Naczk and Shahidi [14], anthocyanins are usually extracted from plant materials with an acidified organic solvent, most commonly methanol. These results were found herein, where methanol/water/acetic acid (70/29.5/0.5, v/v/v) showed the highest ACC for all the analyzed berries. This solvent combination can be destroy the cell membranes, simultaneously dissolving the anthocyanin and stabilizing them [11]. Extractions using acetone and acetone/acetic acid (99.5/0.5, v/v) did not show values of anthocyanins for all the berries studied.

### Antioxidant capacity

Several methods have been employed to evaluate the *in vitro* antioxidant capacity of different plant materials, of which FRAP, DPPH^•^ and ORAC are the most common [23]. Methodologies have different reaction mechanisms, so the results obtained depend on the method used. For this reason, it is recommended to use at least two methods to provide a reliable antioxidant capacity of the sample [24]. In this study, three different methods were used to evaluate the antioxidant capacity of the three fruit extracts: FRAP assay, DPPH^•^ and ORAC assay. The results are provided in Additional file 2.

The antioxidant capacities of the extracts have a strong relationship with the solvent employed, mainly due to the different antioxidant potential of compounds with different polarities [25].

Black mulberry extracts obtained with ethanol/water/acetic acid (50/49.5/0.5, v/v/v) showed the highest antioxidant capacity by FRAP, DPPH^•^ and ORAC methods, with values of 1490.61 mmol Fe^2+^/kg DW, 394.89 mmol TE/ kg DW and 1127.69 mmol TE/ kg DW, respectively. For blackberry extracts, extractions with acetone/water/acetic acid (70/29.5/0.5, v/v/v) showed the highest antioxidant capacity measured by the FRAP method, with a value of 922.28 mmol Fe^2+^/ kg DW. Using the DPPH^•^ and ORAC methods, acetone/water (70/30, v/v) and acetone/water/acetic acid (70/29.5/0.5, v/v/v) presented the highest results and no significant difference between these combinations (p <0.05). For strawberry extracts, extractions with acetone/water (50/50, v/v) presented the highest antioxidant capacity using the FRAP method (499.11 mmol Fe^2+^/kg DW). By DPPH^•^ method the highest results was obtained with acetone (50/50, v/v) and acetone/water/acetic acid (50/49.5/0.5, v/v/v) combination, while the extractions with methanol/water/acetic acid (70/29.5/0.5, v/v/v) and acetone/water/acetic acid (70/29.5/0.5, v/v/v) showed the highest results by ORAC assay.

The lowest antioxidant capacity was obtained using acetone and acetone/acetic acid (99.5/0.5, v/v) for all methods employed and for all the fruits analyzed.

### Effect of the solvent

In the literature, different solvent combinations have been used to extract antioxidants from plant materials such as fruits, vegetables and other foodstuffs. The most widely used solvents for extracting phenolic compounds are water, ethanol, methanol, acetone, and their water mixtures, with acid or not [14,26–28].

The recovery of phenolic compounds is dependent on the solvent used in their extraction and its polarity [12]. This is evident from the TPC and ACC results obtained for the berries as can be seen in Additional file 1. The antioxidant capacities (Additional file 2) of the berry extracts also showed a strong relationship with the solvent employed.

Among the pure solvents, methanol was the most efficient solvent for extraction of antioxidant compounds, followed by water, ethanol and acetone ( see Additional files 1 and 2). These data are agreeing with results reported by Santas et al. [8] who studied two varieties of onion. Phenolic compounds are usually mainly responsible for the antioxidant properties of fruits and vegetables [29] and most of these compounds are classified as hydrophilic antioxidants [30]. This was verified by Wu et al. [31] in which a group of fruits, especially berries, showed higher values for hydrophilic ORAC_FL_ (H-ORAC_FL_) than lipophilic ORAC_FL_ (L-ORAC_FL_). This may explain the results obtained in this work, where methanol and water were the most efficient solvents for the extraction. This could have been due to the better solvation of antioxidant compounds present in fruits as a result of interactions (hydrogen bonds) between the polar sites of the antioxidant molecules and the solvent. Ethanol was less efficient in the extraction of antioxidant compounds than methanol, even if their polarities were similar. This may be due to the low solvation provided by ethanol, probably because of the presence of the ethyl radical that is longer than the methyl radical present in methanol, resulting in a lower solvation of antioxidant molecules. Acetone gave the lowest recovery of antioxidant compounds because of their lower efficiency of solvation, since acetone molecules are only proton acceptors while the other solvents, methanol, ethanol and water, are also proton donors.

Through Additional files 1 and 2 it can be check that the acetone, which is the least efficient solvent when used pure, showed good results when combined with water. This occurred due to increased solvation provided by the presence of water. According to Alothman, Bhat and Karim [12], acetone-water mixtures are good solvent combinations for the extraction of polar antioxidants.

We observed that the addition of acid did not improve the extraction of antioxidant compounds for all the solvent combinations studied (Additional files 1 and 2), which is consistent with other reports [28,32]. In this sense, we observed that this depended on the composition of phenolic compounds in the matrix analyzed.

### Correlation analysis

**Figure 1:**
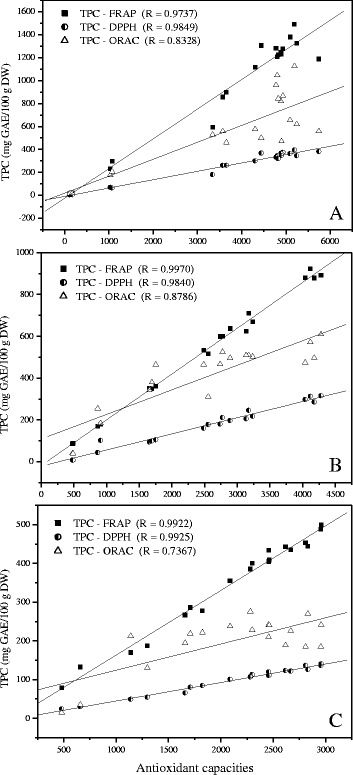
**Relationship between antioxidant capacities and TPC of black mulberry (A), blackberry (B) and strawberry (C).** (TPC: total phenolic content).

Berries contain several phytochemicals and to establish the extent to which polyphenols contribute to the antioxidant properties of the fruits, the Pearson’s correlation (p < 0.05) between the antioxidant capacities (FRAP, DPPH^•^ and ORAC) and TPC was analyzed for all berries (Figure 1).

According to Figure 1, there were significant positive relationships between TPC and antioxidant capacities measured by FRAP, DPPH^•^ and ORAC for all the berries. The high values of Pearson’s correlation coefficients (R) indicated that phenolic compounds are the main contributors to the antioxidant capacities. A positive and significant correlation has also been obtained by other authors [33–35].

The ORAC method was the least correlated with TPC compared to FRAP and DPPH^•^ methods, while all showed a good correlation. This may be due the methods of TPC by Folin-Ciocalteu’s reagent, FRAP and DPPH^•^ methods which involve electron transfer reaction mechanism. This classification can explain the high Pearson’s correlation coefficients shown in Figure 1 because these methods act through the same mechanism. On the other hand, the ORAC method is based on hydrogen atom transfer, this may explain the low value of the correlation coefficient between TPC and ORAC. The same was reported by Gonçalves, Lajolo and Genovese [33] and Isabelle et al. [36].

**Figure 2:**
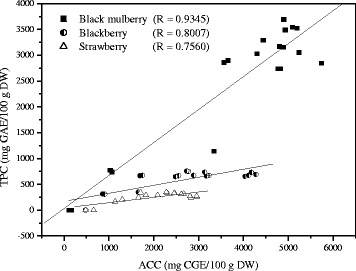
**Relationship between TPC and ACC of berries.** (TPC: total phenolic content; ACC: anthocyanin content)

Anthocyanins are responsible for the red to purple to black pigments found in fruits and vegetables [37] and they are the largest group of water-soluble pigments in the plant kingdom that belong to the class of phenolic compounds [38,39]. From the results shown in Figure 2, can be observed that there were good relationships between TPC and ACC for black mulberry (R = 0.9345), blackberry (R = 0.8007) and strawberry (R = 0.7560).

### Principal component analysis

**Figure 3:**
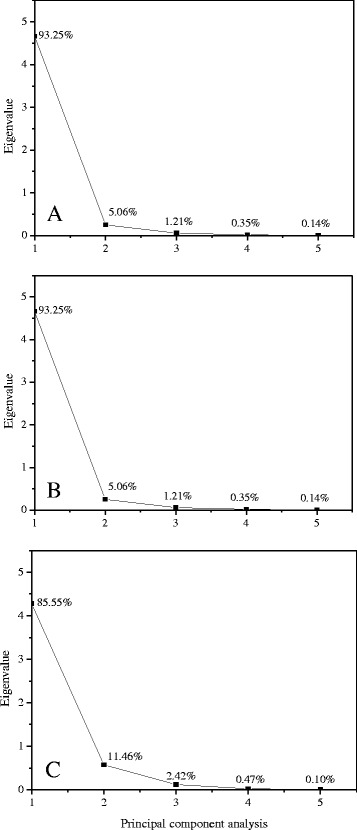
**Eigenvalues of each principal component for black mulberry (A), blackberry (B) and strawberry (C).**

Figure 3 shows two main principal components (PCs) characterized the TPC, ACC e antioxidant capacity (FRAP, ORAC and DPPH^•^) of the nineteen extracts obtained from the black mulberry (Figure 3A), blackberry (Figure 3B) and strawberry (Figure 3C) with a cumulative explained total variance of 98.30%, 98.31% and 97.01%, respectively. For black mulberry, the first principal component (PC1) had the highest eigenvalue of 4.66, and accounted for 93.25% of the variability in the data set. The second PC (PC2) had eigenvalue of 0.25 and accounted for 5.06% of the variance in the data. For strawberry, the PC1 and PC2 had eigenvalues of 4.28 and 0.57, and accounted for 85.55% and 11.46% of the variability in the data set, respectively. For blackberry, the PC1 and PC2 had eigenvalues of 4.56 and 0.36, and accounted for 91.17% and 7.15% of the variability in the data set, respectively. For all berries the remaining three generated PCs yielded progressively smaller eigenvalues and did not explain significant variability in the data (<3% total).

**Figure 4:**
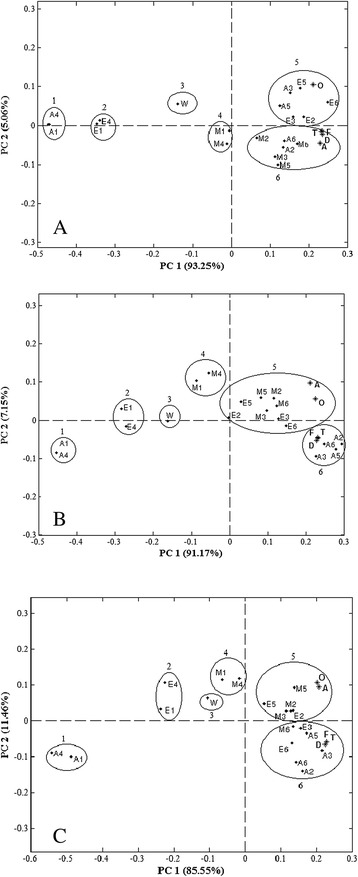
**Biplot (PC1xPC2) of scores and loadings for the PCA of TPC, ACC and antioxidant capacity. (A)** black mulberry, **(B)** blackberry and **(C)** strawberry. (PCA: principal component analysis; TPC: total phenolic content; ACC: anthocyanin content).

Figure 4 shows the biplot of the PC1xPC2 for black mulberry (Figure 4A), blackberry (Figure 4B) and strawberry (Figure 4C). According to these figure six distinct groups were identifiable for all berries. By PC1 the extracts obtained with pure organic solvent, with acid or not (Groups 1, 2 and 4) and with water (Group 3) were separated from the extracts prepared with organic solvent-water mixtures (Groups 5 and 6). This was due to better values presented by groups 5 and 6 for all analysis, compared to other groups. According to Jayaprakasha Singh and Sakariah [40] and Cheng et al. [41], the presence of water increases the permeability of cell tissue and thus, enables better mass transfer by molecular diffusion as well as the recovery of water-soluble bioactive compounds. However extraction with water alone was not as effective as extraction with organic solvent-water mixtures.

Groups 5 and 6 were formed by different extracts according with berry analyzed. For black mulberry (Figure 4A), through PC2 was possible to discriminate between ethanol-water mixtures and methanol–water mixtures. On the other hand, it was not possible to separate acetone-water mixtures. The group 5 formed by E2, E3, E5, E6, A3 and A5, was separated due the highest values of antioxidant capacity by ORAC method, while group 6 formed by M2, M3, M5, M6, A2 and A6 due to the high values of TPC, ACC and antioxidant capacity by DPPH^•^ and FRAP assays.

The Figure 4B showed that PC2 separated acetone-water mixtures (Group 6) from the other organic solvent-water mixtures (Group 5) for blackberry, due to their higher values of TPC and antioxidant capacity by DPPH^•^ and FRAP assays. For strawberry, the PC2 did not show a clear separation between group 5 and 6 (Figure 4C).

## Conclusion

In summary, our results clearly showed that the extraction of phenolic and anthocyanin compounds and their antioxidant capacity is significantly affected by solvent combinations. Black mulberry presented the highest TPC, ACC and antioxidant capacity values. In addition, there was a good correlation between total phenolic content and the antioxidant capacity of the berry extracts. Organic solvent-water mixtures were more efficient in extracting antioxidant compounds than their respective pure organic solvents, with acid or not, and this result agreed with PCA analysis.

## Experimental

### Samples

Fruit samples (~2 kg for each fruit) were acquired from a farm located in south region of Brazil (23°25’30”S and 51°56’20”W). Samples included blackberry (*Rubus ulmifolius*, Rosaceae), black mulberry (*Morus nigra*, Moraceae) and strawberry (*Fragaria* x *ananassa*, Rosaceae). Fresh fruits were washed with tap water, and the edible part of the fruits were separated, cooled and lyophilized. The freeze-dried fruits were milled to obtain fine particles, vacuum packed and stored at −18°C until required for analysis. All the fruits were of eating quality, and were identically selected in terms of shape, size, color and ripening stage.

### Chemical reagents

The reagents used were 2,2-diphenyl-1-picrylhydrazil (DPPH^•^), 6-hydroxy-2,5,7,8-tetramethylchroman-2-carboxylic acid (Trolox), 2,4,6-tris(2-pyridyl)-s-triazine (TPTZ), 2,20-azobis(2-amidinopropane) dihydrochloride (AAPH), fluorescein sodium salt and Folin & Ciocalteu^’^s phenol reagent, all purchased from Sigma-Aldrich. Ferrous sulfate heptahydrate and gallic acid were purchased from Vetec, and sodium carbonate from J.T Baker were also used. All solvents and chemicals were of analytical grade.

### Extraction procedure

The extraction procedure was carried out according to the method of Michiels et al. [28] with slight modifications. Extraction was carried out 1.500 g of freeze-dried berries with 15.0 mL of solvent under magnetic stirring for 1 h in the dark and at room temperature. Then, the solutions were centrifuged for 15 min at 6535 *g* and the supernatant was collected. More than one extraction was carried out with the pellet washed, using 5.0 mL of the same solvent, shaken for 15 min, and centrifuged using the same conditions (6535 *g*, 15 min). After the supernatants were pooled, transferred to a 25-mL volumetric flask and the volume was topped up with the same solvent. The solutions were stored for less than three days, at which time the analyses were carried out.

These solutions were used directly to determine total phenolic content, total anthocyanin content and their antioxidant capacity by FRAP, DPPH^•^ and ORAC assays. Extraction procedures were performed in triplicate.

Three different organic solvents were used for the extraction (methanol, ethanol and acetone) and distilled water (H_2_O). Different organic solvent-water mixtures were also used (70/30, v/v and 50/50, v/v), as well as organic solvents with acetic acid (99.5/0.5, v/v) and organic solvent-water mixtures with acetic acid (70/29.5/0.5, v/v/v and 50/49.5/0.5, v/v/v). Thus each berry was extracted in nineteen solvent combinations (Table 1).

**Table 1:** **Solvent combinations used in the extraction of antioxidant compounds**

**Solvent combination**	**Abbreviation**
Water	W
Methanol	M1
Methanol/water (70/30, v/v)	M2
Methanol/water (50/50, v/v)	M3
Methanol/acetic acid (99.5/0.5, v/v)	M4
Methanol/water/acetic acid (70/29.5/0.5, v/v/v)	M5
Methanol/water/acetic acid (50/49.5/0.5, v/v/v)	M6
Ethanol	E1
Ethanol/water (70/30, v/v)	E2
Ethanol/water (50/50, v/v)	E3
Ethanol/acetic acid (99.5/0.5, v/v)	E4
Ethanol/water/acetic acid (70/29.5/0.5, v/v/v)	E5
Ethanol/water/acetic acid (50/49.5/0.5, v/v/v)	E6
Acetone	A1
Acetone/water (70/30, v/v)	A2
Acetone/water (50/50, v/v)	A3
Acetone/acetic acid (99.5/0.5, v/v)	A4
Acetone/water/acetic acid (70/29.5/0.5, v/v/v)	A5
Acetone/water/acetic acid (50/49.5/0.5, v/v/v)	A6

### Total phenolic content

The total phenolic contents (TPC) of fruits were analyzed according to the Folin-Ciocalteu method [42] with some modifications [43], using gallic acid as the standard. Appropriately diluted extracts (250 μL) were mixed with 250 μL of Folin-Ciocalteu reagent (diluted in distilled water 1:1, v/v), 500 μL of sodium carbonate saturated solution and 4.0 mL of distilled water. The solution was kept in the dark for 25 min, and then centrifuged for 10 min at 1638 *g*. Absorbance at 725 nm was measured in the spectrophotometer (Genesys 10, Thermo Scientific, Madison, USA). Methanolic solutions of gallic acid with concentration of 0 to 250 mg L^−1^ were used for the calibration curve, and results were expressed as g gallic acid equivalents (GAE)/ kg of sample dry weight (DW).

### Anthocyanin content

The anthocyanin content (ACC) was evaluated by the pH differential method [44]. The diluted sample extracts (100 μL) in 25 mmol L^−1^ potassium chloride solution (pH 1.0, 5.0 mL) and 0.4 mol L^−1^ sodium acetate buffer (pH 4.5, 5.0 mL) were measured at 510 and 700 nm, respectively, after 15 min of incubation at 23°C using spectrophotometer. Absorbance variation (A) was calculated as:

1 $$ A={\left({A}_{510} - {A}_{700}\right)}_{pH\ 1.0} - {\left({A}_{510} - {A}_{700}\right)}_{pH\ 4.5} $$

Total anthocyanin content of samples (mg cyanidin3-glucoside L^−1^ of sample extract) was calculated from the following equation:

2 $$ ACC=\frac{A\ x\ M\ x\  DF\ x\ 1000}{\left(\varepsilon\ x\ 1\right)} $$

where A is absorbance value, M is molecular weight (449.2 g mol^−1^), DF is dilution factor (51), and e is the molar absorptivity of cyanidin3-glucoside (26,900 L mol^−1^ cm^−1^). The results were calculated in g cyanidin3-glucoside equivalents (CGE)/ kg of sample dry weight (DW).

### Ferric reducing antioxidant power (FRAP) assay

Antioxidant capacity from the FRAP assay was determined as methodology previously described by Benzie and Strain [45] with modifications by Pulido, Bravo and Saura–Calixto [46]. The FRAP reagent was prepared by mixing acetate buffer (0.3 mol L^−1^, pH 3.6), TPTZ (10.0 mmol L^−1^) and FeCl_3_ (20.0 mmol L^−1^) solutions at the ratio 10:1:1, respectively. 100 μL of diluted sample extracts and 300 μL of distilled water were added to 3.0 mL of the FRAP reagent, which was kept in the dark for 30 min at 37°C. The absorbance was measured in comparison to a blank at 593 nm, using a spectrophotometer (Genesys 10, Thermo Scientific, Madison, USA). Aqueous solutions of known Fe (II) concentrations in the range of 0 to 1500 μmol L^−1^ (FeSO_4_.7H_2_O) were used for the calibration curve and the results were expressed as mmol Fe^2+^/kg DW.

### DPPH^•^ (free radical-scavenging) assay

DPPH^•^ assay was carried out according to the method described by Brand-Williams, Cuvelier and Berset [47] with some modifications [34]. Diluted sample extracts (25 μL) were added to 2.0 mL of 6.25 × 10^−5^ mol L^−1^ DPPH^•^ methanol solution. After gentle mixing and leaving the solutions to stand at room temperature for 30 min, absorbance was measured at 517 nm, using a spectrophotometer (Genesys 10, Thermo Scientific, Madison, USA). Methanol solutions of known Trolox concentrations in the range of 0 to 2000 μmol L^−1^ were used for the calibration curve and the results were expressed as mmol Trolox equivalents (TE)/ kg DW.

### Oxygen radical absorbance capacity (ORAC) assay

ORAC assays were performed according to Ou, Hampsch–Woodill and Prior [48] with modifications by Zulueta et al. [49]. The automated ORAC assay was carried out on a Victor X4 (Perkin–Elmer, USA) 96-well plate reader with fluorescence filters for an excitation wavelength at 485 nm and an emission wavelength at 535 nm. Analyses were conducted in phosphate buffer (75 mmol L^−1^, pH 7.0) at 37°C.

In each well, 150 μL of fluorescein (4.0 nmol L^−1^) and 25 μL of diluted sample extracts, blank (phosphate buffer) or the standard (Trolox solutions at different concentrations) were placed. The plate was then heated to 37°C for 10 min and then 25 μL of AAPH (160 mmol L^−1^) were added. The fluorescence was measured immediately after the addition and measurements were then taken every 1 min for 30 min. To calculate the ORAC value of the samples, the relative fluorescence values at each minute were first generated based on the fluorescence intensity of the samples, blank and standard. Thereafter, th. area under the curve (AUC) of each well was calculated using the following equation:

3 $$ AUC=\left(1 + {f}_1/{f}_0 + {f}_2/{f}_0 + \dots + {f}_i/{f}_0\right) $$

where *f*_0_ is the relative fluorescence at 0 min and *f*_*i*_ is the relative fluorescence at time *i*. The AUC_net_ was calculated by subtracting the AUC of the blank from that of the sample or standard using the equation:

4 $$ AU{C}_{net} = AU{C}_{sample/ standard} - AU{C}_{blank} $$

Then, using a regression equation between AUC_net_ and the Trolox concentration, the final ORAC value was expressed as Trolox equivalents (TE) in mmol/ kg DW.

### Statistical analysis

Results were expressed as means ± standard deviation (SD) and based on dry weight (DW). The results were submitted to variance analysis (ANOVA) and Tukey test (5% probability) using the software Statistica 7.0. The multivariate analysis was performed by applying principal component analysis (PCA) using the MATLAB software version 7.5.0. The data were autoscaled in the pre-processing.

### Additional data file

The following additional data are available with the online version of this paper. Additional file 1 is a table contains the total phenolic content and anthocyanin content of all extracts studied. Additional file 2 contains the antioxidant capacity by DPPH^•^, FRAP and ORAC assays of berry extracts obtained with different solvents.

## Additional files

Additional file 1
**Total phenolic content (TPC) and anthocyanin content (ACC) of berry extracts obtained with different solvents.**
Additional file 2
**Antioxidant capacity of berry extracts obtained with different solvents.**

## Abbreviations

(PCA): Principal component analysis
(PCs): Principal components
(PC1): First principal component
(PC2): Second principal component
(FRAP): Ferric reducing antioxidant power
(DPPH): 2,2-diphenyl-1-picrylhydrazil
(TE): Trolox equivalent
(TPC): Total phenolic content
(GAE): Gallic acid equivalent
(ORAC): Oxygen radical absorbance capacity
(ACC): Anthocyanin content
(CGE): Cyanidin3-glucoside equivalent
(DW): Dry weight
(Trolox): 6-hydroxy-2,5,7,8-tetramethylchroman-2-carboxylic acid
(TPTZ): 2,4,6-tris(2-pyridyl)-s-triazine
(AAPH): 2, 20-azobis(2-amidinopropane) dihydrochloride
(AUC): Area under the curve
(ANOVA): Variance analysis
