# Supplementary material for: Evaluation of solvent effect on the extraction of phenolic compounds and antioxidant capacities from the berries: application of principal component analysis
Source: Chem Cent J. 2014 Aug 22;8:48. doi: 10.1186/s13065-014-0048-1 (PMC4158270; doi:10.1186/s13065-014-0048-1)
Supplement: Additional file 1 — Total phenolic content (TPC) and anthocyanin content (ACC) of berry extracts obtained with different solvents. [file 13065_2014_48_MOESM1_ESM.pdf]

Additional file 1 Total phenolic content (TPC) and anthocyanin content (ACC) of berry extracts obtained with different solvents.

Solvent	Black mulberry		Blackberry		Strawberry	
	TPC (g GAE/ kg DW)	ACC (g CGE/ kg DW)	TPC (g GAE/ kg DW)	ACC (g CGE/ kg DW)	TPC (g GAE/ kg DW)	ACC (g CGE/ kg DW)
W	33.45 ± 0.37^{k}	11.43 ± 0.44^{h}	16.56 ± 0.08^{k}	3.49 ± 0.08^{f}	16.59 ± 0.11^{j}	2.46 ± 0.01^{i}
M1	35.72 ± 0.17^{j}	28.59 ± 0.58^{f}	16.94 ± 0.27^{jk}	6.65 ± 0.06^{de}	18.25 ± 0.11^{h}	2.92 ± 0.00^{f}
M2	43.04 ± 0.43^{i}	30.30 ± 0.25^{e}	27.74 ± 0.20^{g}	7.50 ± 0.07^{ab}	24.63 ± 0.05^{e}	3.32 ± 0.06^{bc}
M3	44.43 ± 0.51^{h}	32.87 ± 0.12^{c}	28.91 ± 0.10^{f}	6.75 ± 0.09^{cd}	23.00 ± 0.14^{f}	3.35 ± 0.10^{abc}
M4	36.56 ± 0.28^{j}	28.95 ± 0.38^{f}	17.54 ± 0.14^{j}	6.73 ± 0.15^{cd}	17.08 ± 0.18^{i}	3.42 ± 0.02^{ab}
M5	48.98 ± 0.35^{de}	36.92 ± 0.08^{a}	27.38 ± 0.43^{g}	7.55 ± 0.10^{a}	22.81 ± 0.16^{f}	3.49 ± 0.02^{a}
M6	50.95 ± 0.20^{c}	35.43 ± 0.25^{b}	31.37 ± 0.24^{e}	7.35± 0.13^{ab}	24.56 ± 0.26^{e}	3.29 ± 0.03^{bcd}
E1	10.33 ± 0.08^{l}	7.72 ± 0.08^{i}	8.65 ± 0.14^{l}	3.11 ± 0.08^{g}	12.99 ± 0.09^{l}	2.05 ± 0.01^{j}
E2	49.33 ± 0.37^{d}	34.87 ± 0.37^{b}	25.56 ± 0.29^{h}	6.67 ± 0.01^{de}	24.56 ± 0.07^{e}	3.24 ± 0.06^{cd}
E3	48.86 ± 0.15^{def}	31.53 ± 0.00^{d}	32.36 ± 0.17^{d}	6.72 ± 0.06^{cd}	26.67 ± 0.09^{c}	3.17 ± 0.05^{de}
E4	10.74 ± 0.03^{l}	7.39 ± 0.00^{i}	9.09 ± 0.06^{l}	3.08 ± 0.14^{g}	11.39 ± 0.05^{m}	1.67 ± 0.01^{l}
E5	48.03 ± 0.17^{fg}	31.69 ± 0.17^{d}	24.87 ± 0.10^{i}	6.45 ± 0.02^{e}	20.88 ± 0.03^{g}	2.92 ± 0.07^{f}
E6	51.92 ± 0.50^{b}	35.24 ± 0.25^{b}	31.75 ± 0.27^{de}	6.62 ± 0.08^{de}	26.17 ± 0.09^{d}	3.09 ± 0.05^{e}
A1	1.16 ± 0.02^{m}	nd	4.79 ± 0.03^{m}	nd	6.59 ± 0.01^{n}	nd
A2	57.44 ± 0.21^{a}	28.41 ± 0.13^{f}	42.81 ± 0.28^{a}	6.92 ± 0.01^{c}	29.55 ± 0.07^{a}	2.53 ± 0.07^{hi}
A3	47.78 ± 0.25^{g}	27.38 ± 0.13^{g}	40.39 ± 0.39^{c}	6.52 ± 0.02^{de}	29.58 ± 0.19^{a}	2.71 ± 0.02^{g}
A4	1.69 ± 0.02^{m}	nd	4.87 ± 0.02^{m}	nd	4.81 ± 0.05^{o}	nd
A5	48.27 ± 0.38^{efg}	27.37 ± 0.47^{g}	41.19 ± 0.15^{b}	6.73 ± 0.04^{cd}	28.31 ± 0.13^{b}	2.45 ± 0.05^{i}
A6	52.42 ± 0.45^{b}	30.52 ± 0.08^{e}	41.78 ± 0.21^{b}	7.30 ± 0.10^{b}	28.08 ± 0.12^{b}	2.67 ± 0.06^{gh}

Mean value ± standard deviation, n = 9. nd: not detected, DW: dry weight, TPC: total phenolic content, GAE: gallic acid equivalents, ACC: anthocyanin content, CGE: cyanidin 3-glucoside equivalents, W: water, M1: methanol, M2: methanol/water (70/30, v/v), M3: methanol/water (50/50, v/v), M4: methanol/acetic acid (99.5/0.5, v/v), M5: methanol/water/acetic acid (70/29.5/0.5, v/v/v), M6: methanol/water/acetic acid (50/49.5/0.5, v/v/v), E1: ethanol, E2: ethanol/water (70/30, v/v), E3: ethanol/water (50/50, v/v), E4: ethanol/acetic acid (99.5/0.5, v/v), E5: ethanol/water/acetic acid (70/29.5/0.5, v/v/v), E6: ethanol/water/acetic acid (50/49.5/0.5, v/v/v), A1: acetone, A2: acetone/water (70/30, v/v), A3: acetone/water (50/50, v/v), A4: acetone/acetic acid (99.5/0.5, v/v), A5: acetone/water/acetic acid (70/29.5/0.5, v/v/v), A6: acetone/water/acetic acid (50/49.5/0.5, v/v/v). Identical superscripts in the same row indicate no significant difference ($p < 0.05$).
