# Supplementary material for: Evaluation of solvent effect on the extraction of phenolic compounds and antioxidant capacities from the berries: application of principal component analysis
Source: Chem Cent J. 2014 Aug 22;8:48. doi: 10.1186/s13065-014-0048-1 (PMC4158270; doi:10.1186/s13065-014-0048-1)
Supplement: Additional file 2 — Antioxidant capacity of berry extracts obtained with different solvents. [file 13065_2014_48_MOESM2_ESM.pdf]

Additional file 2 Antioxidant capacity of berry extracts obtained with different solvents.

Solvent	Black mulberry			Blackberry			Strawberry		
	FRAP (mmol Fe^{2+}/kg DW)	DPPH$^{\bullet}$ (mmol TE/kg DW)	ORAC (mmol TE/kg DW)	FRAP (mmol Fe^{2+}/kg DW)	DPPH$^{\bullet}$ (mmol TE/kg DW)	ORAC (mmol TE/kg DW)	FRAP (mmol Fe^{2+}/kg DW)	DPPH$^{\bullet}$ (mmol TE/kg DW)	ORAC (mmol TE/kg DW)
W	592.50 ± 15.82^{l}	180.55 ± 8.31^{h}	529.35 ± 3.71^{ghi}	350.36 ± 4.87^{i}	94.45 ± 3.22^{k}	341.93 ± 9.11^{de}	266.68 ± 4.35 ^{l}	65.65 ± 1.18^{j}	194.27 ± 3.97^{efg}
M1	856.74 ± 10.21^{k}	260.72 ± 8.00^{g}	558.86 ± 1.60^{fgh}	349.15 ± 1.51^{i}	98.94 ± 2.42^{jk}	379.93 ± 15.80^{d}	278.02 ± 2.46^{k}	84.81 ± 1.88^{h}	221.82 ± 1.02^{bcd}
M2	1115.38 ± 13.91^{i}	299.20 ± 2.73^{f}	576.42 ± 7.06^{fg}	600.09 ± 5.72^{f}	211.93 ± 3.68^{ef}	525.48 ± 13.74^{b}	408.79 ± 1.94^{g}	111.82 ± 1.40^{e}	240.28 ± 5.75^{bc}
M3	1306.51 ± 1.47^{cd}	367.62 ± 5.53^{bc}	500.00 ± 1.74^{hij}	637.08 ± 8.51^{e}	197.21 ± 2.54^{g}	495.82 ± 2.48^{bc}	400.09 ± 2.12^{h}	113.25 ± 0.79^{e}	228.34 ± 5.58^{bcd}
M4	900.09 ± 8.72^{j}	260.59 ± 3.65^{g}	459.84 ± 26.98^{j}	360.19 ± 2.15^{i}	104.62 ± 2.14^{j}	464.78 ± 7.85^{c}	285.87 ± 0.65^{k}	80.16 ± 0.40^{i}	218.87 ± 7.35^{cd}
M5	1230.53 ± 5.65^{fg}	345.02 ± 5.29^{d}	470.68 ± 19.30^{ij}	598.30 ± 2.13^{f}	180.22 ± 4.31^{h}	467.15 ± 15.12^{c}	385.38 ± 0.65^{i}	107.02 ± 2.11^{f}	275.93 ± 11.68^{a}
M6	1380.60 ± 7.03^{b}	363.18 ± 7.29^{c}	655.64 ± 32.47^{e}	623.31 ± 7.21^{e}	205.37 ± 3.26^{fg}	509.27 ± 3.31^{bc}	433.44 ± 1.12^{f}	119.70 ± 1.59^{d}	210.02 ± 3.80^{def}
E1	230.81 ± 3.19^{n}	68.59 ± 0.40^{i}	175.77 ± 1.29^{k}	170.37 ± 2.28^{j}	43.85 ± 1.36^{l}	254.74 ± 6.21^{f}	187.27 ± 1.64^{m}	54.39 ± 0.71^{k}	130.45 ± 8.08^{h}
E2	1276.75 ± 4.45^{de}	373.96 ± 4.55^{bc}	868.43 ± 7.05^{d}	516.02 ± 2.15^{h}	178.06 ± 2.59^{h}	311.12 ± 4.44^{e}	404.69 ± 1.37^{gh}	110.68 ± 1.91^{ef}	242.01 ± 2.38^{b}
E3	1247.27 ± 2.59^{ef}	364.98 ± 9.10^{c}	819.21 ± 9.22^{d}	669.91 ± 8.84^{d}	217.73 ± 2.56^{e}	503.03 ± 6.67^{bc}	435.19 ± 2.66^{ef}	121.62 ± 1.97^{d}	225.93 ± 3.97^{bcd}
E4	294.87 ± 3.22^{m}	63.12 ± 1.22^{i}	206.36 ± 3.56^{k}	179.73 ± 2.94^{j}	102.63 ± 1.58^{jk}	184.07 ± 3.54^{g}	169.77 ± 1.01^{n}	49.22 ± 1.50^{l}	212.57 ± 11.85^{de}
E5	1206.85 ± 4.46^{gh}	344.26 ± 5.53^{d}	1046.98 ± 1.65^{b}	533.78 ± 1.03^{g}	160.30 ± 2.44^{i}	465.72 ± 23.42^{c}	355.02 ± 1.37^{j}	100.75 ± 1.44^{g}	237.66 ± 5.35^{bc}
E6	1490.61 ± 19.25^{a}	394.89 ± 5.20^{a}	1027.69 ± 6.15^{a}	710.48 ± 3.60^{c}	246.17± 2.57^{d}	509.54 ± 0.36^{bc}	442.72 ± 3.88^{de}	123.48 ± 0.00 ^{cd}	188.67 ± 5.29^{fg}
A1	16.32 ± 0.13^{o}	2.65 ± 0.03^{j}	15.27 ± 0.47^{l}	85.06 ± 0.54^{k}	7.27 ± 0.27^{m}	40.71 ± 1.80^{h}	132.50 ± 0.83^{o}	30.19 ± 0.14^{m}	35.43 ± 2.01^{i}
A2	1188.92 ± 10.25^{h}	381.18 ± 1.05^{ab}	559.76 ±12.81^{fgh}	892.42 ± 9.66^{b}	315.42 ± 5.70^{a}	609.45 ± 27.68^{a}	488.13 ± 2.65^{b}	136.24 ± 1.29^{b}	185.06 ± 10.98^{g}
A3	1282.60 ± 17.37^{d}	331.47 ± 1.06^{de}	960.29 ± 51.37^{c}	879.04 ± 2.10^{b}	297.37 ± 5.14^{b}	474.77 ± 3.08^{c}	499.11 ± 0.66^{a}	140.50 ± 0.71^{a}	241.48 ± 1.12^{bc}
A4	20.73 ± 0.21^{o}	3.91 ± 0.10^{j}	16.37 ± 0.02^{l}	88.64 ± 1.00^{k}	6.76 ± 0.12^{m}	39.49 ± 1.36^{h}	78.84 ± 1.11^{p}	24.41 ± 0.68^{n}	15.02 ± 0.73^{i}
A5	1226.37 ± 8.48^{fg}	318.63 ± 4.22^{e}	845.35 ± 50.84^{d}	922.28 ± 10.98^{a}	313.08 ± 3.12^{a}	572.69 ± 20.06^{a}	444.14 ± 5.12^{d}	126.41 ± 1.20^{c}	269.79 ± 11.29^{a}
A6	1327.85 ±12.52^{c}	344.85 ± 4.86^{d}	619.29 ± 23.09^{ef}	878.72 ± 8.73^{b}	285.54 ± 2.45^{c}	497.03 ± 7.53^{bc}	453.03 ± 6.48^{c}	136.73 ± 0.86^{ab}	184.60 ± 1.04^{g}

Mean value ± standard deviation; n = 9. DW: dry weight, TE: trolox equivalent. W: water, M1: methanol, M2: methanol/water (70/30, v/v), M3: methanol/water (50/50, v/v), M4: methanol/acetic acid (99.5/0.5, v/v), M5: methanol/water/acetic acid (70/29.5/0.5, v/v/v), M6: methanol/water/acetic acid (50/49.5/0.5, v/v/v), E1: ethanol, E2: ethanol/water (70/30, v/v), E3: ethanol/water (50/50, v/v), E4: ethanol/acetic acid (99.5/0.5, v/v), E5: ethanol/water/acetic acid (70/29.5/0.5, v/v/v), E6: ethanol/water/acetic acid (50/49.5/0.5, v/v/v), A1: acetone, A2: acetone/water (70/30, v/v), A3: acetone/water (50/50, v/v), A4: acetone/acetic acid (99.5/0.5, v/v), A5: acetone/water/acetic acid (70/29.5/0.5, v/v/v), A6: acetone/water/acetic acid (50/49.5/0.5, v/v/v). Identical superscripts in the same row indicate no significant difference ($p < 0.05$).
